# A 3D In Vitro Cancer Model as a Platform for Nanoparticle Uptake and Imaging Investigations

**DOI:** 10.1002/smll.201400194

**Published:** 2014-07-02

**Authors:** Kate P M Ricketts, Umber Cheema, Agata Nyga, Andrea Castoldi, Chiara Guazzoni, Tarig Magdeldin, Mark Emberton, Adam P Gibson, Gary J Royle, Marilena Loizidou

**Affiliations:** Division of Surgery and Interventional Science, University College London9th Floor Royal Free Campus, Rowland Hill Street, NW3 2PF, London, UK; Department of Medical Physics and Bioengineering, University College LondonMalet Place Engineering Building, WC1E 6BT, London, UK; Dipartimento di Elettronica e Informazione, Politecnico di Milano, Edificio 33, via Rimembranze di Lambrate 14, 20133 Milano, Italy and Instituto Nazionale di Fisica NucleareSezione di Milano, Italy

## Abstract

In order to maximize the potential of nanoparticles (NPs) in cancer imaging and therapy, their mechanisms of interaction with host tissue need to be fully understood. NP uptake is known to be dramatically influenced by the tumor microenvironment, and an imaging platform that could replicate in vivo cellular conditions would make big strides in NP uptake studies. Here, a novel NP uptake platform consisting of a tissue-engineered 3D in vitro cancer model (tumoroid), which mimics the microarchitecture of a solid cancer mass and stroma, is presented. As the tumoroid exhibits fundamental characteristics of solid cancer tissue and its cellular and biochemical parameters are controllable, it provides a real alternative to animal models. Furthermore, an X-ray fluorescence imaging system is developed to demonstrate 3D imaging of GNPs and to determine uptake efficiency within the tumoroid. This platform has implications for optimizing the targeted delivery of NPs to cells to benefit cancer diagnostics and therapy.

## 1. Introduction

Solid cancers (malignant tumors) are composed of cancer foci within a reactive stroma, which is populated by non-cancer cells such as fibroblasts and endothelial cells. The relative constituents vary, resulting in the architectural heterogeneity typical of cancer. This cancer-stromal relationship is easy to observe histopathologically in the tissues. It is less easy to depict when imaging in vivo, either in patients or in animal models, as most imaging modalities (computerized tomography, magnetic resonance and positive emission tomography) do not have the necessary resolution at the micrometer scale. Non-invasive imaging of the tumor microarchitecture and detection of small disseminated disease foci away from the cancer mass boundaries would provide invaluable information that can directly inform treatment choices.

Nanoparticles (NPs) have the potential to act as tumor-specific markers to enhance the resolution of current imaging platforms. Given their relative biologically inert qualities and stability, and unique physicochemical properties, GNPs are good contenders for enhancing imaging sensitivity. They are a reliable contrast medium for use with X-rays due to the high atomic number (*Z*).^1^–^3^ This same attribute can be used to enhance the effects of X-ray radiotherapy^4^–^7^ and proton radiotherapy.^8^ Moreover, GNPs have a modifiable surface that can be used to increase solubility (to travel through the bloodstream) and enhance cell-specific uptake.^9^ Additional coating with polyethylene glycol (PEG), sodium citrate or heparin mitigates any toxicity, prevents initiation of blood coagulation and assists in evading the host immune system.^10^ Further functionalization with cancer specific antibodies can enhance targeting and detection as reported using in vivo models with typical concentrations of the order of 0.01 mgNP/mL taken up by tumors—ten-fold greater compared to concentrations in the surrounding tissue.^11^–^14^ In order to maximize their potential in cancer imaging, NP uptake mechanisms and impact of tumor environmental conditions must be understood.

The cancer-stromal relationship influences how cancer cells uptake exogenous agents such as NPs. The majority of the research on biomarker imaging and NP uptake has been carried out in animal models. Although serving as a useful first step, animal work does not always allow the delineation of the relevant mechanisms at the cellular level and has translated poorly to humans. The emergence of 3D in vitro cancer models^15^ may provide a more direct and reliable method to test novel forms of biomarker-coupled imaging. In this paper we describe novel application of a 3D in vitro cancer model (tumoroid) capable of incorporating GNPs at typical concentrations achieved in vivo. The platform is based on a cancer model we have developed; previous molecular biology studies of the model have been presented to demonstrate biomimicry in terms of tumor growth and progression properties.^16^ Here we present parallel work that demonstrates application of the model as a platform for NP uptake investigations, and as an imaging phantom with ability to inform development of NP imaging technology. It mimics tumor micro-composition, in terms of cell types and spatial positioning to allow specific investigations of NP uptake by cell types found within solid tumors. As a model, it has exciting potential for investigating cancer targeted imaging and therapy and can replace a considerable proportion of work carried out in vivo.

Alongside the cellular model, a technique to measure GNP concentration and distribution is required for uptake investigations. The current gold standard technique for measuring GNP concentration is inductively coupled plasma atomic emission spectroscopy (ICP-AES). However, ICP-AES is not an imaging technique, and digests cells in nitric acid at 110 °C which precludes further analysis.^17^ As well as a clinical need for non-destructive measurement methods, there is also a requirement for a GNP imaging technology that is sensitive to sufficiently low concentrations. Current imaging technologies are lacking in this respect, with the lowest measurable concentration of 0.05 mgAu/mL using micro-computed tomography.^12^ We have developed a novel non-destructive X-ray fluorescence (XRF) technique that can be used to improve on NP imaging capabilities lacking in current systems. This technique achieves sensitivity more than an order of magnitude greater than other reported techniques down to GNP concentrations of 0.005 mgAu/mL.^18^ XRF enables a greater penetration depth than optical techniques (being a higher energy modality) and offers potential for simultaneous imaging of multiple NP compositions. Our approach shows potential in both quantification of and sensitivity to NP concentrations typically found in tumors.^18^

## 2. Results

### 2.1. GNP Uptake

**Figure 1:**
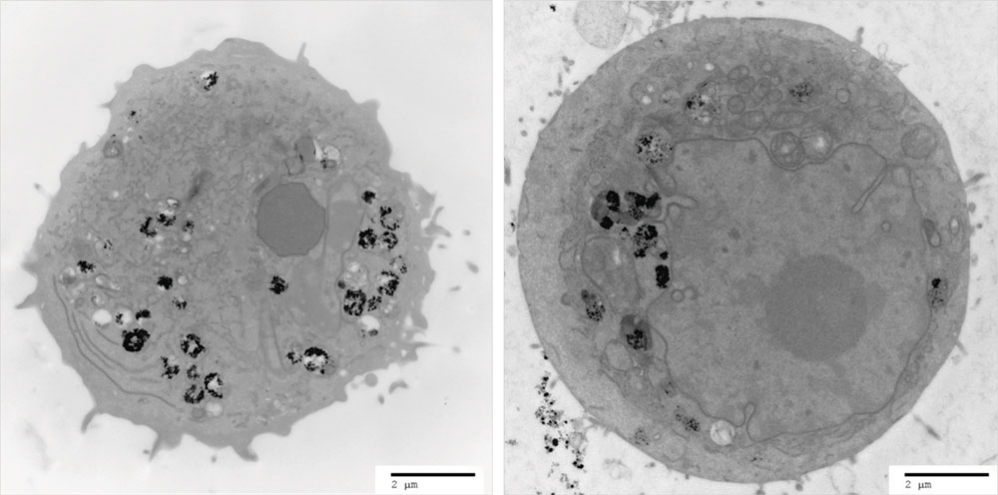
TEM image of a 3T3 fibroblast (left) and an HT29 cancer cell (right) incubated with 1.9 nm GNPs (incubation time 24 h). The cells appear viable; the dark appearance of the cytoplasm indicates presence of ground substances and a healthy state; organelles required for healthy cell functioning are present (such as the nucleus, endoplasmic reticulum and mitochondria). GNPs (black round spheres) appear either dispersed or as aggregates, mostly encapsulated in cytoplasmic vesicles.

**Figure 2:**
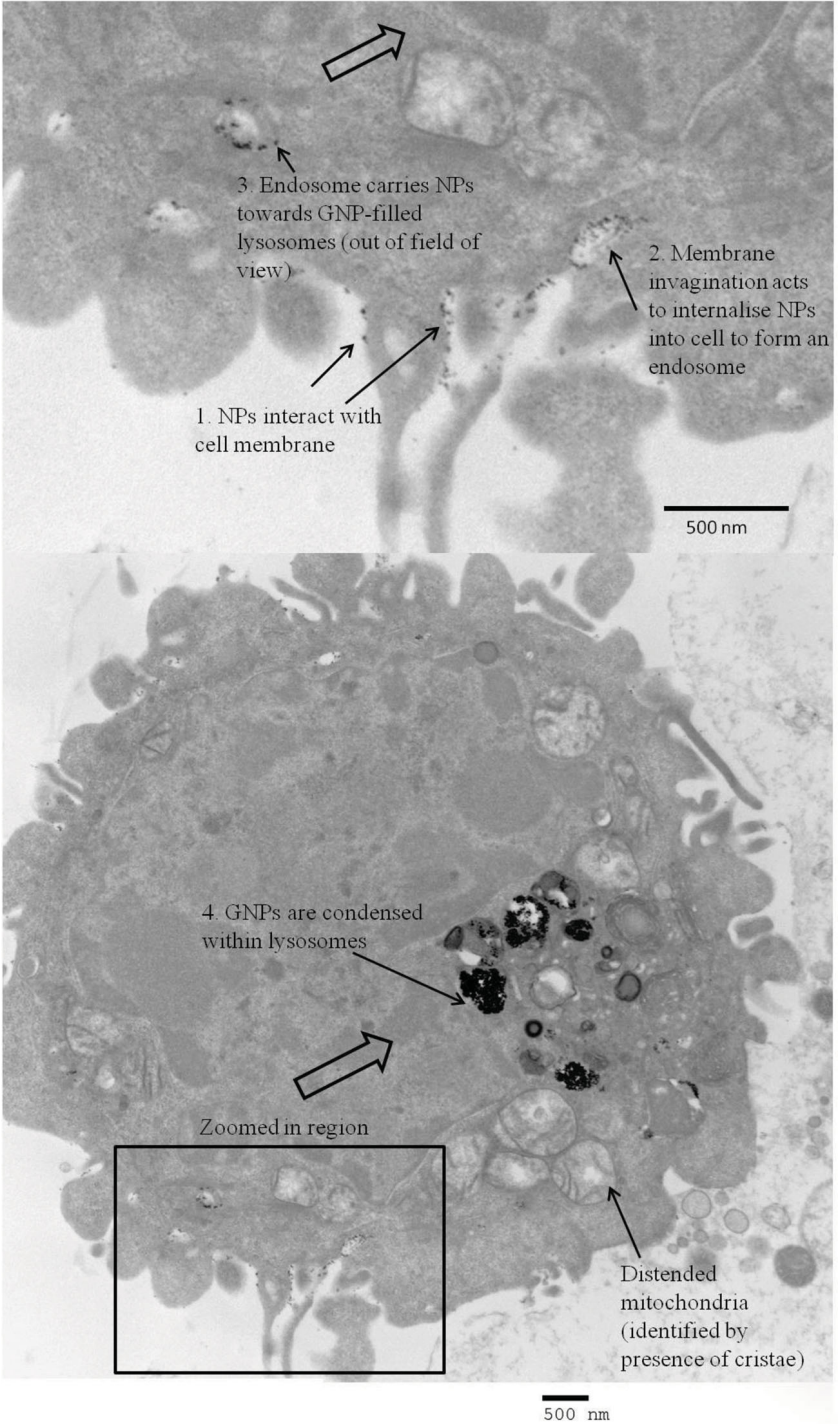
TEM image of the cell membrane surface of an HT29 cancer cell incubated with 1.9 nm GNPs (incubation time 24 h). The image has captured each step of endocytosis: 1) GNP-membrane interaction, 2) membrane invagination to take up GNPs in to the cell, and 3) resulting endosome transports GNPs towards the lysosomes (4).

The first step of tumoroid production was to initiate GNP uptake within cell lines of each tumoroid component (artificial cancer mass: HT29 colorectal cancer cells, stromal component: 3T3 murine fibroblasts). Uptake of GNPs of diameter 1.9 nm was achieved by both cell lines (incubation time 24 h) as demonstrated by TEM (**Figure** **1**), and appeared to be through endocytosis (captured in **Figure** **2**). An incubation time of 24 h was found to give optimum GNP uptake in order to maximize GNP imaging signal per cell. It was observed that incubation times greater than 24 h were associated with signs of cell stress and apoptosis (up to 10% cell death at incubation time 48 h). X-ray microanalysis confirmed gold presence within the cells; this involved a TEM technique that bombarded the sample with electrons and detected the emitted X-rays (the energy of emission being characteristic to elemental composition).

### 2.2. GNP Uptake Measurement using X-Ray Fluorescence

Before complex tumoroids were constructed, uncompressed 3D constructs were manufactured in order to test GNP uptake over a range of incubation doses. The following results enabled relation of GNP incubation dose with uptake concentrations for each cell line; this data was subsequently used to inform controlled engineering of GNP concentration within each tumoroid component (ACM and stroma).

#### 2.2.1. Measured XRF Spectra

**Figure 3:**
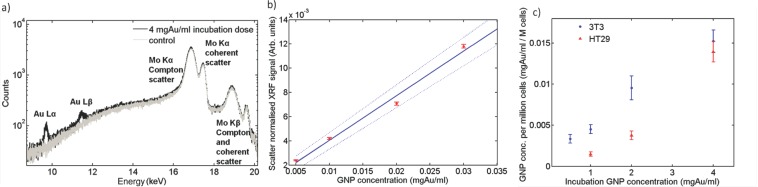
Quantitative XRF measurement of GNP concentration within 3D constructs. a) Measured spectra of 4 mgAu/mL GNP incubation dose uncompressed HT29 cellular construct and 0 mgAu/mL (control) acquired over 40 mins; b) Calibration curve relating XRF signal to GNP concentration. The XRF signal has been normalized to the Compton peak and acquisition time.^20^ A weighted linear fit (solid line) and boundary levels (dashed lines) are shown. The boundary levels were fit to fully include 95% of the data points and their error bars; the latter calculated using Poisson statistics. Measurements were made over a range of known GNP concentration solutions; c) GNP concentrations measured in 3T3 and HT29 3D uncompressed cell-populated collagen gel constructs over a range of initial incubation doses. The boundary levels of (b) were used to determine the error on each sample GNP concentration measurement, which was then added in quadrature to the error in the XRF measurement resulting from statistical fluctuations.

To confirm sensitivity to gold the XRF system was used to obtain emission spectra for two 3D uncompressed HT29 cellular constructs (incubated with 4 mgAu/mL and 0 mgAu/mL (control); **Figure** **3**a). Typical measurement times ranged from 40 min for high concentration samples to 400 min for the lowest concentration. The cell samples incubated with gold present a spectrum with the characteristic gold fluorescence lines, compared with the control samples which showed no gold present.

#### 2.2.2. Calibration Curve

XRF analysis was performed on a range of known GNP concentration solutions in order to determine the actual GNP concentration present in the 3D constructs. The resulting calibration curve ranged from 0.005 mgAu/mL to 0.03 mgAu/mL (Figure 3b).

#### 2.2.3. Gold Concentration per Sample

Figure 3c displays the measured GNP concentration found for the 3T3 and HT29 3D uncompressed constructs. Repeats at the incubation dose 4 mgAu/mL gave the same final GNP concentration within measurement error. The XRF signal from the sample incubated with 4 mgAu/mL was four times greater than that incubated at 1 mgAu/mL (Figure 3c). TEM images qualitatively corroborated the XRF technique's findings that uptake was proportional to initial GNP dose. Attaining a known GNP uptake was shown to be repeatable and reproducible well within the standard errors of measurement. Percentage uptake of initial incubation GNP dose per million cells as measured by a non-destructive XRF technique was found to range between 0.3–0.5% and 0.1–0.3% for the 3T3 and HT29 cell constructs respectively. An order of magnitude estimate of the number of GNPs taken up per cell was undertaken for the highest measured GNP uptake (the 3T3 sample given an incubation dose of 4 mgAu/mL) as follows. The mass of gold per cell was estimated: A GNP concentration of ≈0.015 mgAu/mL was measured for this sample using XRF analysis (Figure 3c); cell number was normalized to 1 million cells per mL. This corresponds to 0.015 ngAu per cell. The mass of one 1.9 nm GNP was estimated: Each NP was assumed to be a solid sphere of gold of calculated volume 4.2 nm^3^. The volume was multiplied by the density of gold (19.32 g/cm^3^) to give an estimated mass of 7 × 10^−11^ ng per NP. For this sample we inferred from XRF emissions that ≈2 × 10^8^ GNPs were present per 3T3 cell.

### 2.3. Tumoroid Production

We implemented a 3D in vitro tissue-engineering technique to create a 3D model that incorporates NP cellular uptake: where the basic premise was to create a dense artificial cancer mass (ACM) comprised of HT29 colorectal cancer cells set within a dense collagen scaffold, surrounded by uncompressed collagen populated by non-cancer cells, in this case fibroblasts to mimic cancer stroma.^19^

**Figure 4:**
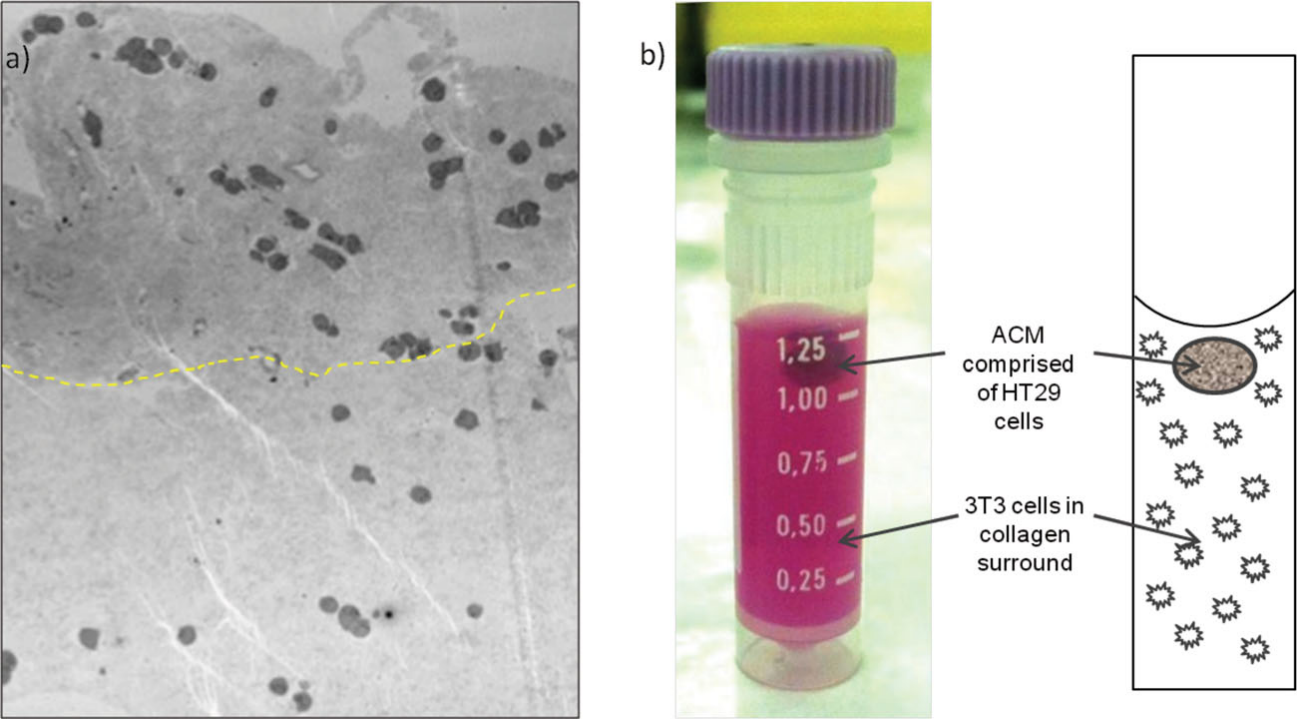
Tumoroid consisting of an artificial cancer mass (ACM):stromal surrounding GNP concentration ratio 5:1, the ACM of approximate concentration 0.02 mgAu/mL. a) Microscopic appearance (TEM) of tumoroid photographed in (b). The boundary between ACM and stroma is evident as a difference in collagen density, the greatest difference in collagen greyscale mapped as a dashed line to estimate the cancer/stromal boundary; collagen is more dense within the ACM (above dashed line) and stromal collagen less dense (below dashed line). GNP containing vesicles are visible within the cells.

Successful manufacture of a 3D tumoroid (full construct dimensions 8 mm diameter, 18 mm height; ACM diameter 4 mm) was demonstrated with 0.7 × 10^6^ HT29 cells pre-incubated with 5 mgAu/mL for 24 h (compressed ACM) and 6.3 × 10^6^ 3T3 fibroblasts pre-incubated with 2 mgAu/mL for 24 h (uncompressed stromal component). The incubation doses were chosen to engineer an ACM GNP concentration of 0.02 mgAu/mL and GNP concentration ratios of 5:1 between the ACM and surrounding stroma to resemble conditions achieved in vivo. The engineered GNP-loaded tumoroid is displayed in **Figure** **4**.

### 2.4. 3D XRF Imaging of Tumoroid

**Figure 5:**
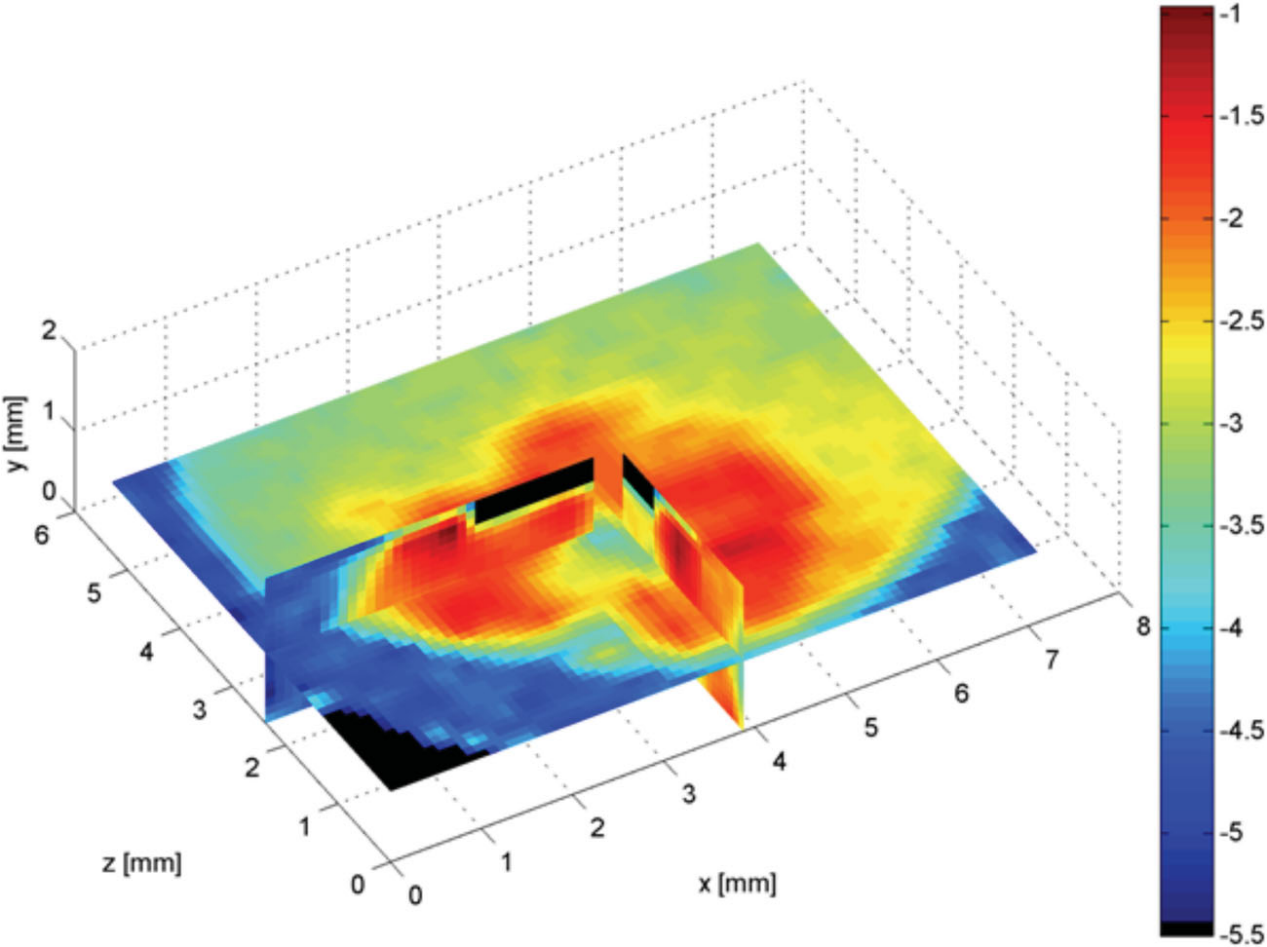
XRF image of three different cross sections of a tumoroid composed of an ACM and surrounding 3T3-embedded collagen gel with a challenging GNP concentration ratio 5:1 between the ACM and surrounding. The blue-to-red colour scale presents gold concentration (red representing the highest gold concentration).

3D XRF imaging was performed of several image slices of the tumoroid, with a partial reconstruction displayed in **Figure** **5**. All component details were clearly visible with a delineated ACM emitting at five times more than the surrounding cellular stroma. The image presented in Figure 5 was taken at the Diamond synchrotron source, UK. The log scale amplified recognition of the tumor region (red corresponding to highest gold concentration), the surrounding background (green corresponding to a lower gold concentration) and the plastic container (blue corresponding to absence of gold) in which the tumoroid was set. A control sample with no GNP incubation yielded no gold signal and so the ACM could not be distinguished from the surround.

## 3. Discussion

### 3.1. Summary of Results

We have presented a 3D in vitro cancer model (tumoroid) that was successfully used as a platform to image GNP uptake and distribution at concentrations achieved in human tissue. The tumoroid was engineered to have distinct tumor and stromal compartments mimicking the microarchitecture of solid cancers and developed as a more controllable replacement to small-animal models in order to assess NP delivery to tissue under controlled conditions.

The uptake of 1.9 nm GNPs into the cells used for the uncompressed single cell type 3D constructs and the more complex tumoroids was passive and largely related to the cell surface area, with 3T3 cells being larger than HT29 cells (data not shown). It is likely that this passive uptake was facilitated by protein adsorption on to the GNP surface resulting in a protein corona from serum proteins found in the growth medium, which brings them in prolonged contact with the cell membrane and promotes endocytosis.^20^ There is huge potential for active targeting through conjugation of the NPs to antibodies that target tumor biomarkers and provide functional information as to the cellular characteristics of the tumor; for example for use in mapping hypoxia.

Dose dependent uptake was demonstrated at incubation times of 24 h. Signs of cytotoxicity were observed for longer incubation time. However, evidence for the cytotoxicity of GNPs is equivocal and requires further study.^11^,^21^,^22^

To our knowledge, no other tissue engineered in vitro alternative exists as an imaging platform for research in to imaging biomarkers. Our construct is biomimetic in terms of cell composition and spatial orientation (cancer mass surrounded by collagen matrix and stromal cells). In particular, by using plastic compression, we have increased the density of cell-seeded collagen type I hydrogels to ≈7% (w/v), which aims to approach densities found in vivo (generally accepted as >10%). Cell viability was retained in cells embedded in the scaffold during compression (results not shown); a previous study showed that the standard compression method results in only a 10% reduction in cell viability.^23^ Using a tissue engineering approach allows a high degree of modulation and reproducibility which is needed to probe how NP uptake is influenced by controlled introduction of further cell types and incorporation of different matrix components according to designed spatial positioning within specific local matrix densities. To the authors’ knowledge, the latter and its importance in cell uptake behavior within tissues is not addressed by any other existing in vitro cancer model.

Furthermore we have presented a novel quantitative imaging module (XRF) that is sensitive to GNP concentration and distribution and can perform non-destructive imaging of bulk 3D samples, the latter imperative for NP uptake studies. To summarise the important novel characteristics of our imager, achieved through it being purpose designed and custom built, we can now meet the needs of NP-imaging that are required by the community and yet to be met in the literature: i) sensitivity to low NP concentrations typically found in vivo (our system is an order of magnitude more sensitive to others reported, with a detection limit of 0.005 mgAu/mL), ii) ability to quantify NP concentration over a 3D matrix: our high energy technique can measure bulk 3D volumes at depth (1–2 cm) currently unachievable by NP imaging techniques, iii) contains 3D positional information of NPs (with spatial resolution better than other technologies due to implementation of polycapillary optics), iv) does not require destruction of the sample unlike gold standard TEM and ICP-AES. In addition the module has potential for multiparametric imaging (simultaneous measurement of multiple NP types). We used both XRF imaging and the reference standard of TEM to observe GNP uptake and distribution in our 3D tumoroids. The use of GNPs as a contrast agent enabled micrometer scale tumor detail to be imaged as can be observed in Figure 5. This result demonstrates clinical potential of GNP-XRF imaging; tumors are not homogeneous, regularly shaped tissues and as such require an imaging technique to fully characterise the cell types and distributions within them to fully inform therapy regimens.

Our XRF technique was used to perform an order of magnitude estimate of the number of GNPs taken up per cell. At the highest given GNP dose of 4 mgAu/mL we estimated that ≈2 × 10^8^ GNPs were present per 3T3 cell. This number of NPs per cell is in keeping with previous reports; one study quantified NP uptake per cell at a rate that was four orders of magnitude lower than our own (3000 per cell) for HeLa cells given an incubation dose four orders of magnitude lower (0.14 μgAu/mL) with 14 nm GNPs.^20^ A different NP size, and GNP stabilized with sodium citrate and different cell lines renders the results not directly comparable, but indicates that our estimates of number of GNPs per cell is appropriate and accurate.

### 3.2. Clinical Implications

We have demonstrated the ability of imaging micrometer scale tumor detail using GNPs as a contrast agent and an XRF technique sensitive to GNP concentration and distribution. This holds great potential in the development of platform technology, which relates to: i) testing active targeting of GNPs to tumors for a range of tumor characteristics under controlled conditions using our model as a platform; NP XRF can be used to determine microstructural and functional signals of a range of biomarkers within the tumoroids, ii) harvesting and growth of cancer cells from a patient within our tumoroids, we can test the tumor characteristics, signalling and response to therapies on a patient-by-patient basis, delivering potential for true personalized medicine, iii) the development of a higher energy XRF system for potential to work towards GNP XRF in vivo imaging of tumors to guide therapy; the possibility of clinical translation from synchrotron to bench-top source has been demonstrated previously,^18^ iv) the ability to map specific tumor characteristics such as hypoxia can be used to inform cancer therapy regimens, for example, radiotherapy dose escalation to hypoxic regions, and v) NP XRF imaging enables capability to detect small clusters of infiltrating cancer cells commonly missed by current imaging modalities.

GNPs were selected in this study to demonstrate the concept of our platform for nanoparticle uptake and imaging studies as they are widely used within the community due to their clinical viability and are heavily reported within the literature; however our uptake and imaging platform is not limited solely to gold and will be used in future studies for uptake investigation of a range of clinically relevant metallic nanoparticles.

### 3.3. Future Research

This work provides the basic protocol for assessing biofunctionalized NP uptake in 3D in vitro models through the development of tumoroids; we shall extend the current work, which involved NP uptake in monolayer cultures to investigate the cellular uptake of NPs in 3D cultures to represent more realistic in vivo conditions representative of the clinical situation. We also aim to make the model more sophisticated in terms of adding flow components to simulate lymphatic drainage and vasculature leakage.

The tumoroids provide a more controllable replacement to small animal models in order to assess imaging and treatment regimes under modifiable tumor and stromal conditions. For example, investigations of the dose enhancing power of GNPs in radiotherapy will be undertaken using the models. Tumoroid technology has the potential to escalate progress in NP applications in cancer imaging and provision of personalized cancer therapy, providing a platform to inform targeted drug delivery and uptake, and to assess the effectiveness of therapy at the cellular level. We have been developing in parallel a model for investigations in to tumor growth and progression and for therapeutic screening.^16^ This model has demonstrated the formation of a hypoxic core at the centre of the ACM and expression of vascular endothelial growth factor (VEGF) by cancer cells at the stromal boundary. The level of hypoxia is controllable and dependent on the cell and extracellular matrix (ECM) density. We aim to implement such tissue engineering strategies to investigate the effectiveness of NPs targeted for hypoxia. We will also use the tumoroids to investigate multi-biomarker imaging where different molecular targeting biomarkers can be tagged to different element NPs each of which will give a specific XRF signal and allow the sophisticated mapping of disease or response to treatment.

## 4. Conclusions

A 3D in vitro colorectal tumoroid, which mimics the microarchitecture of solid cancers incorporating GNPs at typical concentrations achieved in vivo, was developed in order to provide a nanoparticle uptake platform in order to develop nanoparticle imaging and therapy studies under controlled, variable tumor and stromal conditions.

Production of the tumoroids involved two stages during their development: i) effecting GNP uptake in cells, and ii) embedding the cells in a 3D scaffold. The former stage was successfully achieved with relative ease, thought to be passively facilitated by protein adsorption on to the GNP surface of serum proteins found in the growth media. Future work will look at different culture media to see if different protein corona on the surface of GNPs has an influence on cell uptake and warrants further investigation. Percentage uptake of initial incubation GNP dose per million cells was found to range between 0.3–0.5% and 0.1–0.3% for the 3T3 and HT29 cell constructs respectively. In an in vivo situation tumor cells will take up more GNPs than healthy cells due to leaky tumor vasculature and poor lymphatic drainage at tumor sites. The differential uptake of GNPs between cancer and stromal cells may be improved through functionalisation using specific antibodies, growth factors, and targeting peptides to meet the overall eventual aim of this project in using GNPs as contrast agents to image the distribution of bio-parameters of a tumor. TEM images qualitatively corroborated the XRF technique's findings that uptake was proportional to initial GNP dose, and captured the nanoparticle internalisation process of endocytosis.

3D-XRF imaging successfully measured a challenging GNP concentration ratio of 5:1 between the ACM and stroma of the tumoroid and all component details could clearly be seen. The current model will be extended in the future from GNP incubation in 2D culture towards 3D culture incubation techniques. Here our aim is to mimic the tumor microenvironment through platform technology to determine how cell-cell communication, cell-ECM interactions, ECM and cell density, and outside physical and mechanical forces found under physiological conditions such as shear stress and fluid flow, will affect and influence the uptake of nanoparticles in cells, making use of the non-destructive XRF imaging technique to map the time course of GNP distributions within the model, which will have direct implications in personalized medicine and patient healthcare.

## 5. Experimental Section

*Gold Nanoparticles*: GNPs with a 1.9 nm gold core and water-soluble organic shell (Aurovist, Nanoprobes Inc., USA) were suspended in phosphate buffered saline (PBS) and/or cell culture media to form 10 mgAu/mL stock solution. GNP size quantification of these commercially available GNPs was previously measured through TEM analysis and found to have a mean particle diameter of 2.2 ± 0.2 nm (≈70% of particles ranged between 1.5 nm and 2.5 nm).^24^

*Cell Culture and GNP Incubation*: The human colon adenocarcinoma cell line HT29 (passages [p]:30-42) and the mouse embryonic fibroblast cell line 3T3 (p:28-32) (ECACC, Sigma-Aldrich, Dorset, UK) were cultured routinely under aseptic humidified conditions at 37 °C in 5% CO_2_/95% air, in Hepes buffered Dulbecco's Modified Eagle Medium (DMEM plus 1000 mg glucose/L and l-glutamine, Sigma-Aldrich); and supplemented with foetal calf serum (10% FCS, 5129, First Link UK Ltd, Birmingham UK) and penicillin/streptomycin (1% P/S, GIBCO, Invitrogen, Paisley UK). For GNP experiments, cells were grown in 25 cm^2^ flasks to 75% confluence and incubated with GNPs (0.5–5 mgAu/mL) in order to i) relate GNP incubation concentration with cellular uptake concentration for each cell line, and ii) to inform engineering of tumoroids to allow control of GNP concentration in each component (ACM and stroma). Incubation was performed under a range of incubation times up to 48 h, under routine conditions, to ascertain an incubation time for optimized GNP uptake within the limits of cell viability. After incubation, a strict protocol was followed to ensure reproducibility and maximum cell harvesting. The cells were washed with PBS (2 × 3 min), PBS-EDTA (1 × 2 min) and enzymatically detached using 0.5 mL of 1 mg/mL Trypsin in PBS-EDTA (1 min). Cells were resuspended in 5 mL media, 50 μL were removed for counting (haemocytometer) and the remaining were centrifuged (400*g*, 5 min). Cells were resuspended in a final 0.1 mL or 0.4 mL of media, as appropriate to be used for manufacturing of 3D in vitro constructs.

*Manufacture of 3D Uncompressed Cellular Constructs*: 3D tissue culture constructs were engineered using HT29 cancer cells and 3T3 fibroblasts. All cells had taken up GNPs. The formation of collagen type 1 lattices involves fibrillogenesis—a thermodynamically driven fibril aggregation following neutralization of native collagen solution. Resultant collagen hydrogels consist mainly (> 99%) of water and are therefore of a much lower collagen density than that found in vivo. We mixed collagen type I (rat-tail collagen type I, protein concentration 2.035 mg/mL in 0.6% acetic acid, First Link UK Ltd.) and minimum essential medium (MEM, 10X with Earle's Salts, without l-glutamine, sodium bicarbonate, GIBCO 21430, Invitrogen) and adjusted the pH to 7-7.7 (NaOH: 1 m, 5 m), as judged by indicator colour change. GNP-containing cells (either HT29 or 3T3) were immediately added and the mixture micro-pipetted to ensure even cell dispersion. Collagen:medium:cell volume ratios were 8:1:1. The mixture (1 mL) was transferred to a polypropylene container (inner/outer diameter 8/9 mm, height 45 mm, 1.5 mL volume, Nunc), and the gel allowed to set in a humidified incubator, 5% CO2/95% air at 37 °C for a minimum of 30 min.

*Manufacture of Tumoroid*: A 3D in vitro tissue-engineering technique was implemented to create a 3D model that enables NP cellular uptake: where the basic premise was to create a dense artificial cancer mass (ACM), surrounded by uncompressed collagen populated by non-cancer cells, in this case fibroblasts to mimic cancer stroma.^19^ Briefly, for the ACM, collagen type I and minimum essential medium (MEM) were mixed and the pH adjusted to 7-7.7 (described above). GNP-containing HT29 cells were immediately added to the mixture using a micro-pipette, and poured into a cuboidal mould resting on a nylon mesh which was in turn placed on top of a 165 μm stainless steel mesh and absorbent paper and allowed to set into a gel at room temperature for 30 mins.^19^ The gel was compacted using a standard plastic compression protocol modified from^18^ (compression under a 73.55 g load, for 5 mins). The gel was turned over and the process repeated to reduce cell concentration gradients resulting from directional fluid flow. This compression results in approximately a 16–fold increase in collagen density compared to uncompressed gels giving a final collagen density for the ACM of 6.7% (w/v).^25^ Collagen:MEM:cell volume ratios were 8:1:1 with an overall, pre-compression volume of 4 mL. The dense ACM cuboid gel was divided in to ≈4 mm segments and each segment nested into a 1 mL uncompressed collagen gel populated with GNP-containing 3T3 fibroblasts (prepared as above) before placing in the incubator and allowed to set. Full construct dimensions were 8 mm in diameter, 18 mm in height, and an ACM diameter of 4 mm. The construct was incubated for at least 30 mins before fixation with 10% gluteraldehyde (in PBS) for 24 h at room temperature. The 30 minute incubation times were not sufficient for cell-mediated gel contraction, which habitually starts within a few hours of incubation and can continue to over a week.^26^

*Transmission Electron Microscopy (TEM)*: Fixed 3D culture constructs were dissected using a scalpel blade in to cubes < 2 mm in thickness, washed in PBS and postfixed using 1% osmium tetroxide/1.5% potassium ferricyanide (BDH, Leicester, UK) for 1.5 h; washed in distilled water and dehydrated through a degrading ethanol series (30% to 100%). They were then placed in 50% alcohol/50% Lemix epoxy resin (TAAB Laboratories Equipment Ltd, Reading, UK) mixture overnight (18 h), and the following day infiltrated with 100% Lemix resin for 2 d and finally embedded in fresh Lemix resin and polymerized at 70°C overnight (18 h). Ultrathin sections were cut using a diamond knife (Diatome) and collected on 300HS, 3.05 mm copper grids (Gilder). Sections were stained with saturated uranyl acetate in 50% ethanol (TAAB) for four minutes followed by Reynold's lead citrate for 5 min (BDH). Sections were viewed using a Philips CM120 TEM and photographed with an AMT Digital Camera (Deben UK).

*X-Ray Fluorescence Technique*: A technique based on inducing X-ray fluorescence (XRF) in GNPs was developed to enable non-destructive quantitative assessment of the GNPs incorporated into the 3D constructs.^18^ Here we have extended this 1D measurement technique to enable step and shoot 3D imaging of bulk samples.^27^ The system comprised a laminar incident excitation X-ray beam to excite the GNPs to fluoresce, in conjunction with an energy-resolving 10 mm^2^ silicon drift detector (SDD) coupled to a slightly focusing polycapillary optic which allowed collection and 3D mapping of fluorescence emissions.^28^,^29^ The magnitude of the XRF signal characteristic to gold was proportional to the concentration of GNPs in the measurement volume. To calibrate, 1 mL GNP solutions were used at 0.005–0.04 mgAu/mL; the concentration range covered the range of GNP concentrations retained by the cell preparations.

*GNP Uptake Measurement using X-Ray Fluorescence*: Cell cultures (HT29, 3T3) were incubated with GNPs (0.5–4 mgAu/mL) for 24 h and used for manufacture of 1 mL 3D uncompressed cellular constructs (as above). A maximum of 5 × 10^6^ cells/mL were embedded into the collagen gel. XRF measurement using a clinically available bench-top molybdenum X-ray tube was used to determine the internalized GNP uptake efficiency over a range of concentrations.

*XRF Imaging of Tumoroid*: Measurements were made at the Diamond Light Source, Oxford, UK. The incident energy was optimized for gold XRF excitation, set to 15 keV (above the edge for gold L-fluorescence of 11.9 keV and 13.7 keV for Lα/Lβ_2_ and Lβ_1_ fluorescence respectively), a compromise between optimum excitation and minimization of the impact of the Compton shoulder background on the gold fluorescence line. The beam, sample centre and SDD-optic module were aligned. A CCD camera located behind the sample was used to monitor the beam-sample alignment. An ionization chamber was used to monitor beam intensity in order to normalize the data. The sample was then scanned in both the *x*-, *y*-, and *z*-directions to build up a pixellated image. The full energy spectrum was obtained at each point. GNP images were reconstructed off-line by computing the integral counts under the gold *Lα* and *Lβ* fluorescence peaks.
